# Supplementary material for: The UHPLC-QTOF-MS Phenolic Profiling and Activity of Cydonia oblonga Mill. Reveals a Promising Nutraceutical Potential
Source: Foods. 2021 May 28;10(6):1230. doi: 10.3390/foods10061230 (PMC8228880; doi:10.3390/foods10061230)
Supplement: Supplementary file 1 [file foods-10-01230-s001.zip › foods-1235546-SI/Supplementary material/Supplementary Table S3.pdf]

Supplementary Table S3: Semi-quantitative values for the main phenolic sub-classes by Ultra-High-Performance Liquid Chromatography Quadrupole Time-of-Flight (UHPLC-QTOF) mass spectrometry of *Cydonia oblonga Mill.* methanolic extracts (*i.e.*, whole fruit, leaf and stem). The values of total flavonoids content were obtained by summing flavones, flavanols and flavonols, while the total phenolics content were obtained by summing total flavonoids content, lignans, low molecular weight, phenolic acids and stilbenes.

	**Anthocyanins** *(Cyanidin eq.)*	**Flavones** *(Luteolin eq.)*	**Flavanols** *(Catechin eq.)*	**Flavonols** *(Quercetin eq.)*	**Lignans** *(Sesamin eq.)*	**LMW** *(Tyrosol eq.)*	**Phenolic acids** *(Ferulic acids eq.)*	**Stilbenes** *(Resveratrol eq.)*	**Total flavonoids content**	**Total phenolics content**
**Leaf**	4.55±0.22^{a}	52.15±0.23^{a}	9.94±0.45^{a}	11.50±0.46^{a}	72.32±3.16^{b}	68.07±6.51^{a}	29.24±4.70^{a}	13.25±1.43^{a}	78.14±1.37^{a}	261.02±15.10^{a}
**Whole fruits**	1.06±0.05^{a}	21.69±0.60^{c}	1.69±0.03^{b}	2.91±0.72^{b}	42.67±1.96^{c}	43.85±2.97^{c}	33.07±1.55^{a}	11.79±0.58^{a}	27.34±1.40^{c}	158.73±8.74^{b}
**Stem**	3.58±0.22^{a}	29.98±2.50^{b}	7.31±0.27^{a}	4.00±0.02^{b}	127.72±10.09^{a}	58.16±6.77^{b}	25.85±3.52^{b}	6.29±0.51^{b}	44.87±3.01^{b}	262.89±23.10^{a}

Values are presented as the mean ± standard deviation (n = 3). The results are expressed as mg Equivalents (Eq.)/g dry matter. The different superscript letters differ in $p<0.05$, Duncan's post-hoc. LMW: Lower molecular weight phenolics.
